# Supplementary material for: Different geomorphic processes control suspended sediment and bedload export from glaciers
Source: Nat Commun. 2025 Jul 1;16:6005. doi: 10.1038/s41467-025-60776-4 (PMC12219753; doi:10.1038/s41467-025-60776-4)
Supplement: Supplementary file 1 — Supplementary Information [file 41467_2025_60776_MOESM1_ESM.pdf]

Supplement: Different hydro-geomorphic processes control bedload and suspended sediment export from glaciers

Ian Delaney[1], Fred Lardet[1,2], Matt Jenkin[1], Davide Mancini[1], and Stuart N. Lane[1].

[1]Institut des Dynamique de la Surface Terrestre (IDYST), Université de Lausanne Bâtiment Géopolis, CH-1015 Lausanne, Switzerland.

[2]Now at: Laboratory of Catchment Hydrology and Geomorphology, School of Architecture, Civil and Environmental Engineering, Ecole Polytechnique Fédérale de Lausanne, Route des Ronquos 86, CH-1951 Sion, Switzerland.

1 Tables

Table S1: Model parameters used for inversion

Name	Symbol	Units	Parameter Range	Sampling Distribution
Sediment production rate*	$\dot{\varepsilon}$	$\mathrm{mm\,a^{-1}}$	$(10^{-5}, 3.5)$	Uniform
Initial till condition	H_0	m	(0.0001, .0025)	Uniform
Hooke angle	β	rad	$(\frac{\pi}{15}, \pi)$	Uniform
Friction factor	fi	–	$(3, 55)$	Uniform

* Equivalent to 3.17×10^{-15} to 1.11×10^{-10} with units $\mathrm{m\,s^{-1}}$

Table S2: Model variables and parameters.

Name	Symbol	Value	Units
	**General**		
Water discharge (instantaneous)	Q_w		$\mathrm{m^3\,s^{-1}}$
Sediment discharge (observation; by volume)	Q_o		$\mathrm{m^3\,s^{-1}}$
Gravitational constant	g	9.81	$\mathrm{m\,s^{-2}}$
Density of water	ρ_w	1000	$\mathrm{kg\,m^{-3}}$
Density of ice	ρ_i	917	$\mathrm{kg\,m^{-3}}$
Density of bedrock and sediment	ρ_b	2650	$\mathrm{kg\,m^{-3}}$
Model misfit (sediment volume)	ξ	-	-
	**Subglacial model**		
Sediment discharge	Q		$\mathrm{m^3\,s^{-1}}$
Sediment discharge capacity, *SSL*, *BL*	$\widehat{Q}_s$, $\widehat{Q}_b$		$\mathrm{m^3\,s^{-1}}$
Width of channel floor	$\widehat{w}$		m
Channel cross-sectional area	S		$\mathrm{m^2}$
Hydraulic diameter	D_h		m
Hydraulic head	Δh		m
Hydraulic gradient	$\Psi = \frac{\Delta h}{l}$		$\mathrm{mm^{-1}}$
Water velocity	$v = \frac{Q_w}{S}$		$\mathrm{m\,s^{-1}}$
Water shear stress	τ		Pa
Hooke angle of channel	β	-	rad
Constant 1	C_1	2.2×10^{-5}	$\mathrm{m^{-1}}$
Constant 2	C_2	3.7×10^{-13}	$\mathrm{m}^{-n}\, s^{-1}$
Latent heat of fusion	L	333.5	$\mathrm{kJ\,kg^{-1}}$
Pressure melting coefficient	c_t	7.5×10^{-8}	$\mathrm{K\,Pa^{-1}}$
Specific heat capacity of water	c_p	4180	$\mathrm{J\,kg^{-1}K^{-1}}$
Ice flow constant	A	5.3×10^{-24}	$\mathrm{Pa}^{-n}\, s^{-1}$
Ice flow exponent	n	3	(−)
Darcy-Weisbach friction factor	f_i	-	-
Till height erosion limit	H_{max}	0.05	m
Till source term	$\dot{m}$		$\mathrm{m\,s^{-1}}$
Sediment production rate	$\dot{\varepsilon}$		$\mathrm{m\,s^{-1}}$
Till layer height	H		m
Mean sediment grain size	D_{50}	0.002, 0.078	m
Glacier length	l	6000	m
Glacier width	w	410	m
Ice thickness	h_{ice}	125	m
Ice thickness at proglacial area	h_p	0	m
	**Numerical parameters**		
Solver tolerance (relative)	reltol	10×10^{-8}	-
Solver tolerance (absolute)	abstol	10×10^{-8}	m
Maximum timestep	dtmax	21600 (6)	s (hr)
Minimum timestep	dtmin	1	s
Initial Conditions	S_0, H_0	-	$\mathrm{m^2}$, m
Number of accepted runs		2×10^6	-

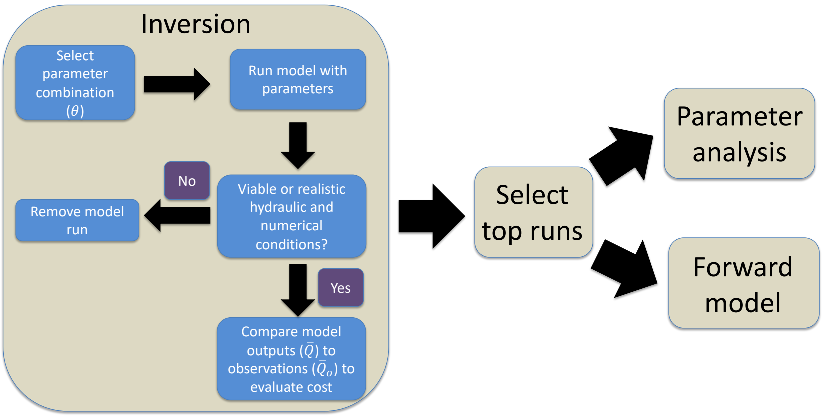

Figure S1: Flow chart of steps in modeling framework.

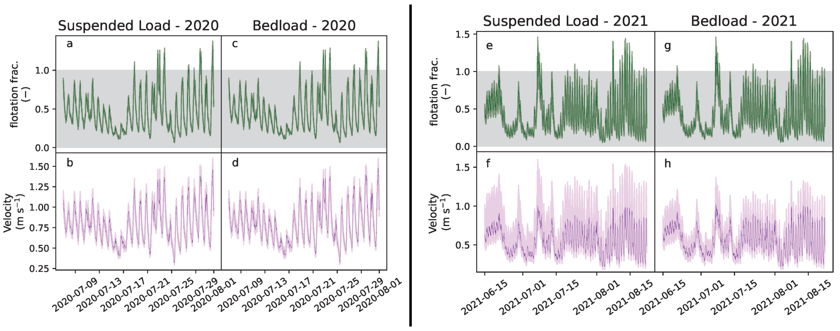

Figure S2: Model outputs of flotation fractions (a, b, c, d) and water velocities (e, f, g, h) for the selected parameter combinations. Shaded regions of a-d show flotation fractions less than 1.

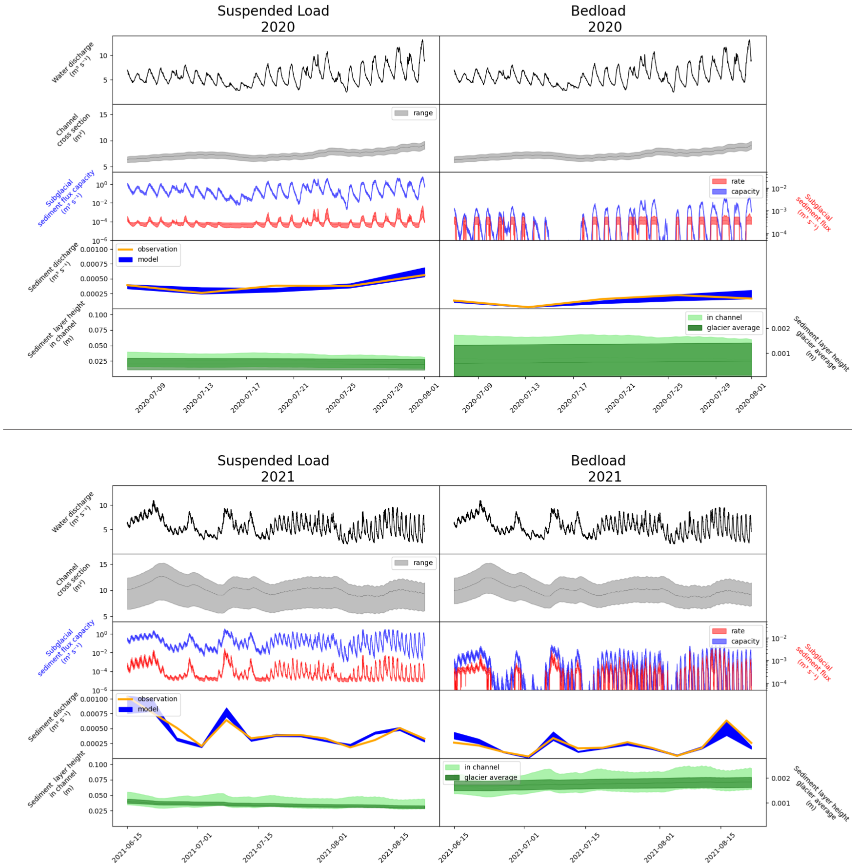

Figure S3: As Figure 3 in text, with channel cross-sectional area and channel and subglacial sediment layer thickness averaged over the glacier (dark green) and averaged over the evolving channel width (light green).

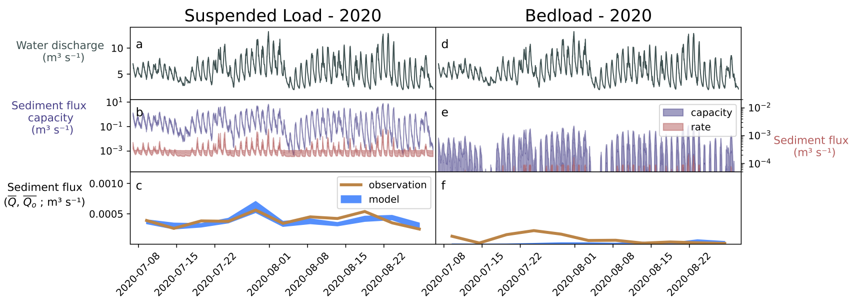

Figure S4: As Figure 3 in text, for complete season of 2020 for both SSL and BL. Note poor model performance in BL.

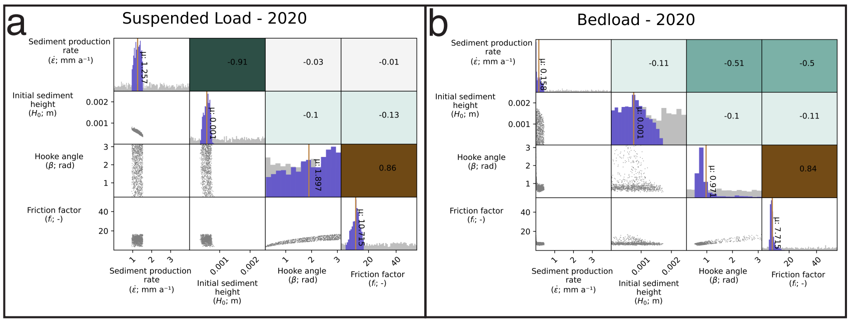

Figure S5: As Figure 4 in text, for complete season of 2020 for both SSL and BL.
